# Pentraxin-3 Levels Relate to the Wells Score and Prognosis in Patients with Acute Pulmonary Embolism

**DOI:** 10.1155/2019/2324515

**Published:** 2019-03-12

**Authors:** Haotian Yang, Jun Zhang, Ying Huan, Yawei Xu, Rong Guo

**Affiliations:** ^1^Department of Cardiology, Shanghai Tenth People's Hospital, Shanghai 200072, China; ^2^Nanjing Medical University, Nanjing, Jiangsu 211166, China; ^3^Tianjin Medical University, Tianjin 300060, China

## Abstract

**Objective:**

To investigate the value of the PTX-3 test in evaluating the prognosis of acute pulmonary embolism (APE).

**Method:**

117 APE patients were selected and divided into two groups according to plasma PTX-3 levels, including the group in which PTX − 3 ≥ 3.0 ng/mL (*n* = 42) and the group in which PTX − 3 < 3.0 ng/mL (*n* = 75). Patients were stratified into high-risk, medium-risk, and low-risk groups according to the Wells scores, and the PTX-3 levels were compared among the groups. Patients had been followed-up as well.

**Results:**

According to the Wells scores, 11 patients were classified as high-risk (9.4%) and 68 were medium-risk (58.1%), while 38 were low-risk (32.5%). The PTX-3 levels in different risk groups were statistically different (all *P* < 0.05). During the follow-up period, 6 deaths occurred in the group with elevated PTX-3 (≥3.0 ng/mL), while 2 deaths occurred in the group with nonelevated PTX-3 (<3.0 ng/mL). The difference between the two groups was statistically significant (*P* < 0.01). 13 patients were hospitalized due to recurrent pulmonary embolism, of which 12 were in the group with elevated PTX-3 (≥3.0 ng/mL), while 1 patient was in the group with nonelevated PTX-3 (<3.0 ng/mL). The difference was statistically significant (*P* < 0.01).

**Conclusion:**

The plasma PTX-3 level in APE patients is correlated with PE risk stratification. There is a significant correlation between PTX-3 levels and PE-related cardiac deaths, as well as the prognosis of recurrent PE. PTX-3 can be used as a clinical indicator of PE prognosis.

## 1. Introduction

Pulmonary embolism (PE) is a clinical, pathological, and physiological syndrome, in which the pulmonary arteries are blocked by a variety of endogenous or exogenous emboli, which is manifested as pulmonary circulation dysfunction in most cases [1]. In the US, PE is the third most common cardiovascular disease, with an incidence after coronary artery disease and high blood pressure. Studies indicate that 1 out of 1000 hospitalized patients suffers from PE, with a mortality rate as high as 25%-30% if left untreated [2]. In recent years, as the knowledge on PE deepens, both PE diagnosis and the treatment have advanced significantly. However, it remains challenging in clinical practices to perform accurate PE diagnosis and risk stratification and to develop proper treatment plans in accordance with both [3]. Although numerous studies have proved the importance and practical roles of various predicative scoring in PE diagnosis [4, 5], we continue to explore new biomarkers to facilitate PE diagnosis and treatment and to evaluate or predict the risk of PE.

Inflammation plays an important role in the occurrence and development of venous thromboembolism [6, 7]. Currently, multiple studies have confirmed the correlation between inflammatory mediators and PE [8–10]. Pentraxin-3 (PTX-3) is a pentameric structural protein containing 381 amino acids, i.e., pentraxin. Currently, the role that PTX-3 plays in cardiovascular disease has been subjected to extensive studies. However, not many studies on how the plasma PTX-3 level in PE patients correlates with PE and its implication in PE prognosis were carried out. Our hypothesis is that the plasma PTX-3 level in PE patients is helpful in the diagnosis of PE and is closely related to PE prognosis. Therefore, we attempt to investigate the correlation of PE diagnosis and prognostic determination with plasma PTX-3 levels in PE patients upon hospitalization.

## 2. Materials and Methods

### 2.1. Clinical Information

The inclusion criteria are the following: (1) clinical presentation and lab and imaging results in accordance with the diagnostic criteria defined in “Diagnosis and Treatment of Pulmonary Thromboembolism Commonly Agreed by Experts in China,” issued by the Pulmonary Vascular Disease Group of Chinese Society of Cardiology in 2010; (2) high compliance and availability for follow-ups; and (3) complete clinical information.

The exclusion criteria are the following: (1) ineligibility to the above diagnostic criteria, (2) lost to follow-up, (3) other chronic comorbidities (malignant tumors, severe infection, impaired liver and kidney function, etc.), and (4) previously confirmed PE and rehospitalization for further screening or treatment without evidence of PE recurrence.

### 2.2. Sample Collection, Processing, and Detection

Venous blood samples were drawn in all patients upon hospitalization. Sample collection and processing requirements were conducted as follows: 5 mL of venous blood was drawn from subjects and centrifuged for 10 min at 8000g/min. The plasma was isolated and stored in a freezer at -80°C. PTX-3 concentration was determined after all the samples were collected using the enzyme-linked immunosorbent assay. Pentraxin-3 was detected using commercial ELISA kits (Perseus Proteomics, Tokyo, Japan), following the manufacturer's instructions.

The Wells score criteria are as follows: (1) malignancy (1 point), (2) immobilization/surgery within 4 weeks (1.5 points), (3) hemoptysis (1 point), (4) history of deep vein thrombosis or PE (1.5 points), (5) heart rate > 100/min (1.5 points), (6) alternative diagnosis other than PE (3 points), and (7) clinically suspected DVT (3 points). According to clinical significance, PE risk < 2 is classified as low risk, PE risk of 2-6 is classified as medium risk, and PE risk >6 is high risk.

### 2.3. Follow-Ups, Medication, and Monitoring

All patients were taking oral warfarin after discharge, and the dose was adjusted according to the prothrombin time (PT), so that the PT was prolonged to 2-3 times the international normalized ratio (INR). All patients underwent “face-to-face” follow-up to find out the usage of warfarin, adverse reactions to the drug, and clinical outcomes, which include cardiac deaths and PE recurrence (worsening of clinical symptoms, plasma D-dimer > 0.5 *μ*g/mL, and expansion of embolism as shown in pulmonary artery CTA), and to monitor INR.

All subjects were followed up for 12 months after release. This study was approved by the ethics committee of the hospital, and all subjects had signed the informed consent before the study enrollment.

### 2.4. Statistical Analysis

Measurement data that follows a normal distribution was shown as mean ± SD, while data following nonnormal distribution was shown as median (M). The measurement data was analyzed using the *t*-test and rank-sum test, while the enumeration data was analyzed with the chi-squared test. A logistic regression model was used in multivariate analysis. SPSS 13.0 software was used in statistical analysis, and a difference of *P* < 0.05 was considered statistically significant.

## 3. Results

### 3.1. Establishment of Cut-Off Values and Baseline Data

157 PE patients were screened and hospitalized in our heart center between January 2012 and December 2016. 40 PE patients were excluded due to loss to follow-up (*n* = 2), malignant tumors (*n* = 10), severe infections (*n* = 8), major organ dysfunction (*n* = 11), and other conditions (*n* = 9). 117 patients were selected finally, including 78 males and 39 females, aged between 27 and 89 (63.2 ± 11.5) years. Pulmonary artery CTA was the diagnostic test for PE patients, in which PE was set at 1 and non-PE was set at 0. The ROC curve was drawn by plotting sensitivity as a function of 1 − specificity. The AUC_ROC_ was 0.863, and the 95% confidence interval was 0.80–0.95. Based on our previous research experience [11], by setting the threshold at 3.0 ng/mL, both the sensitivity and specificity of PTX-3 were relatively high (84.3% and 88.7%, respectively). Therefore, in this study, 3.0 ng/mL of plasma PTX-3 was used as the threshold to divide the patients into two groups, including the group with elevated PTX-3 (*n* = 42) and group with nonelevated PTX-3 (*n* = 75). The detailed general comparison between the two groups of patients is shown in Table 1.

### 3.2. Wells Score and Risk Stratification

Among the 117 patients, the lowest Wells score was 0, whereas the highest score was 11.5, and the average was 3.3. The SD was 2.5. According to the scores, 11 patients were identified as high-risk (9.4%), 68 were medium-risk (58.1%), and 38 were low-risk (32.5%).

### 3.3. PTX-3 Levels

**Figure 1:**
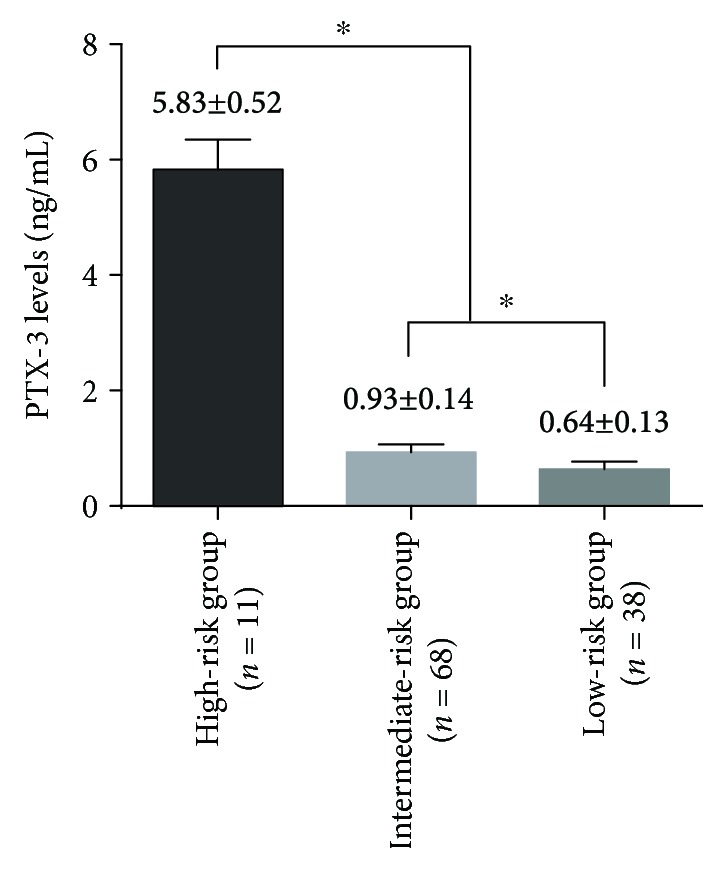
Plasma S100A1 levels in the groups with different risk stratification. ^∗^*P* < 0.05.

The plasma PTX-3 level in the 117 patients was 1.30 ± 1.83 ng/mL. The plasma PTX-3 levels of high-risk, medium-risk, and low-risk patients were 5.83 ± 0.52, 0.93 ± 0.14, and 0.64 ± 0.13 ng/mL, respectively (Figure 1). The difference of the three PTX-3 levels was statistically significant (*P* < 0.05). The differences between either two of the three levels were statistically significant (all *P* < 0.05).

### 3.4. Follow-Ups and Outcomes

**Figure 2:**
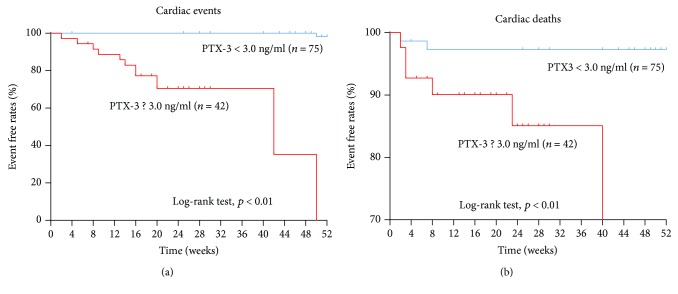
Kaplan-Meier survival analysis. (a) The difference of cardiac deaths is statistically significant between the PTX-3-low and PTX-3-high groups (*P* < 0.01). (b) The difference of cardiac events is statistically significant between the PTX-3-low and PTX-3-high groups (*P* < 0.01).

115 out of the 117 patients completed the follow-up. The remaining 3 patients could not be reached due to phone number changes. The average follow-up period was 36.3 ± 17.4 weeks. Among the 115 followed patients, 8 cardiac deaths occurred between 1 week and 6 months after the discharge. Among them, 6 deaths occurred in the group with elevated PTX-3 (≥3.0 ng/mL), while 2 deaths occurred in the group with nonelevated PTX-3 (<3.0 ng/mL). This difference is statistically significant (*P* < 0.01, Figure 2(a)). 13 patients were hospitalized for PE recurrence, of which 12 were from the group with elevated PTX-3 (≥3.0 ng/mL), while 1 was from the group with nonelevated PTX-3 (<3.0 ng/mL). This difference is statistically significant (*P* < 0.01, Figure 2(b)).

## 4. Discussion

As APE patients differed in clinical severity, the treatment strategies and prognosis also differed [12]. Certain patients with rapid disease progression and comorbidities, such as hypotensive shock, right-sided heart failure, and death, required interventions such as cardiopulmonary resuscitation, thrombolysis, tracheal intubation, and intravenous vasopressors [13–15]. In recent years, cardiac biomarkers have been widely used clinically, such as BNP, cTNT, and D-dimer, an indicator of coagulation function [16, 17]. In this study, plasma PTX-3 in PE patients was measured and prognosis was evaluated in the early stage according to patients' PTX-3 levels as a guide for the clinical treatment plan. The main findings in this study include the following: (1) the PTX-3 level is correlated with the Wells score to some degree and (2) PTX-3 in PE patients is significantly related to the prognosis of the disease, which can be used as an indicator for prognostic evaluations.

As PE lacks specific clinical presentation, it can be easily confused as other cardiopulmonary diseases, such as the coronary artery disease mentioned, acute COPD exacerbation, pulmonary infection, tumor, and pleural effusion. Due to the difficulties in early diagnosis, PE has a high rate of misdiagnosis and missed diagnoses, resulting in a high rate of disability and death [18]. Therefore, fast and effective early screening strategies are crucial in PE diagnosis. The Wells score was proposed much early and is widely used in clinical practice. As the source of the Wells score is the patient information obtained in hospitals and clinics, arterial blood gas test results are not needed. However, one of the criteria that has high scores is subjective, which is the likelihood of alternative diagnosis to PE. As it is difficult to standardize this variable, this significantly affects the evaluation of the patient [19, 20]. A comparative study of the Wells score and the revised Geneva score by Klok et al. in 300 suspected PE patients revealed that the difference between the two scores was not statistically significant, in the PE incidence among the unlikely, somewhat likely, and highly likely groups. The difference of the area under the ROC curve was also statistically insignificant. Therefore, the Wells score and the revised Geneva score were considered to have the same predicative value in PE [21]. Thus, the Wells score was used in this study to evaluate the risk stratification of PE patients.

PTX-3 was not only the first identified classic pentraxin but also an acute-phase reaction protein in inflammation, which is capable of binding to multiple soluble receptors and ligands and is involved in multiple biological effects, such as immune defense, inflammation, and atherosclerosis [22]. PTX-3 belongs to the pentraxin protein family along with short pentraxins, such as C-reactive protein (CRP) [23]. PTX-3 synthesis is mainly induced by proinflammatory cytokines, such as tumor necrosis factors and interlukin-1, in vascular endothelial cells and macrophages. Compared to CRP, PTX-3 is a better reflector of vascular inflammatory conditions [24]. Multiple clinical studies had confirmed that PTX-3 is closely related to the occurrence and prognosis of vulnerable plaques in atherosclerosis and acute coronary syndrome [25–29].

There have been extensive recent studies investigating the roles of PTX-3 in vascular disease and angiogenesis [30, 31]. PTX-3 is produced not only by vascular smooth muscle cells and endothelial cells with localized inflammation but also by macrophages and monocytes in invasive lesions [23]. It was reported that PTX-3 could be used as a biomarker in multiple types of pulmonary hypertension (PH), including idiopathic PH, connective tissue disease-associated PH, PH associated with congenital heart disease, and chronic thromboembolic PH [32–34]. However, it remains unclear whether PTX-3 can be used as a clinical biomarker for APE. In this article, the feasibility of using PTX-3 in PE risk stratification and clinical prognosis was investigated. It is discovered that the plasma PTX-3 level in PE patients is positively correlated with the risk level. In addition, in this study, the patients from the group with elevated PTX-3 had significantly higher risks of cardiac deaths and recurring PE, demonstrating a significant value in clinical practice.

Nonetheless, the flaws and insufficiencies in this study must be acknowledged. This study is a retrospective analysis, and the patient number is limited. Therefore, the final result needs to be validated by a multicenter, prospective study with a large sample number. In addition, this study failed to investigate the effects of interventions on the plasma biomarker levels.

## 5. Conclusion

Plasma PTX-3 level in APE patients is correlated with PE risk stratification. PTX-3 level is significantly correlated with the prognosis of PE-related cardiac deaths and recurring PE and can be used as a clinical indicator in PE prognostic determination.

## Figures and Tables

**Table 1:** Baseline characteristics of enrolled patients.

	PTX − 3 ≥ 3.0 ng/mL (*n* = 42)	PTX − 3 < 3.0 ng/mL (*n* = 75)	*P* value
Male/female	30/12	48/27	0.54
Age	59.6 ± 8.5	59.4 ± 10.5	0.89
BMI (m/kg^2^)	24.2 ± 2.6	24.4 ± 2.3	0.62
Tobacco use (*n*, %)	26 (61.9%)	26 (34.7)	0.006
Hypertension (*n*, %)	19 (45.2%)	45 (60.0%)	0.18
Diabetes (*n*, %)	23 (54.8%)	38 (50.7%)	0.70
Dyslipidemia (*n*, %)	27 (64.3%)	44 (58.7%)	0.69
Coronary artery disease (*n*, %)	5 (11.9%)	12 (16.0%)	0.59
Atrial fibrillation (*n*, %)	9 (21.4%)	4 (5.3%)	0.013
Cerebrovascular disease (*n*, %)	16 (38.1%)	28 (37.3%)	0.93
DVT (*n*, %)	24 (57.1%)	23 (30.7%)	0.006
Pulmonary disease (*n*, %)	23 (54.8%)	47 (62.7%)	0.44
Tumor (*n*, %)	8 (19.0%)	11 (14.6%)	0.61
Recent operation history (*n*, %)	10 (23.8%)	8 (10.6%)	0.067
Bone fracture (*n*, %)	7 (16.7%)	12 (16.0%)	0.92
Trauma (*n*, %)	4 (9.5%)	9 (12.0%)	0.77
Heart rate (beat/min)	83.2 ± 18.1	76.8 ± 13.1	0.03
SBP (mmHg)	124.7 ± 22.6	135.9 ± 20.5	0.007
DBP (mmHg)	68.4 ± 11.7	73.7 ± 10.5	0.013
Shock or hypotension (*n*, %)	10 (23.8%)	4 (5.3%)	0.006
Right heart insufficiency (*n*, %)	20 (47.6%)	13 (17.3%)	0.001
Respiratory failure (*n*, %)	30 (71.4%)	26 (34.6%)	0.00
cTNT (ng/mL)	1.43 ± 1.01	0.98 ± 0.82	0.01
NT-proBNP (pg/mL)	2196.8 ± 1784.7	742.0 ± 509.3	0.00
D-dimer (*μ*g/mL)	7.78 ± 4.34	4.35 ± 2.95	0.00
GFR (mL/min)	85.2 ± 10.3	87.5 ± 10.7	0.29

BMI: body mass index; DVT: deep vein thrombosis; SBP: systolic blood pressure; DBP: diastolic blood pressure; cTNT: cardiac troponin T; NT-proBNP: N-terminal pro-B-type natriuretic peptide; GFR: glomerular filtration rate.

## Data Availability

All data generated or analyzed during this study are included in this published article.
